# Efficacy and Safety of Fluconazole Mucoadhesive Patches in Human Immunodeficiency Virus-Related Oral Candidiasis

**DOI:** 10.1177/23259582241299014

**Published:** 2024-12-05

**Authors:** Srikanth Deenadayalan, Ashok Shenoy, Ashwin Kamath, Mahalaxmi Rathnanand, Sheetal Ullal, Nandita Shenoy

**Affiliations:** 1 Medical Affairs, Novo Nordisk, A/S Soborg R&D Centre, Soborg, Denmark; 2 Department of Pharmacology, Kasturba Medical College Mangalore, Manipal Academy of Higher Education, Manipal, Karnataka, India; 3 Department of Pharmaceutics, Manipal College of Pharmaceutical Sciences, Manipal Academy of Higher Education, Manipal, Karnataka, India; 4 Department of Oral Medicine and Radiology, Manipal College of Dental Sciences Mangalore, Manipal Academy of Higher Education, Manipal, Karnataka, India

**Keywords:** fluconazole, localized delivery, mucocutaneous candidiasis, bioadhesive ﬁlm, good health, quality of life

## Abstract

**Background:**

Opportunistic fungal infections like oral candidiasis account for a significant amount of morbidity in HIV disease and an indicator of immune suppression. Fluconazole is a broad-spectrum antifungal agent that has been extensively used in the management of oral, candidiasis. Highly efficacious fluconazole is also known to have systemic toxicity due to high drug interaction and hence the present study focuses on the formulation of bioadhesive film as a controlled release carrier for fluconazole.

**Materials and Methods:**

Patients were randomised, using a computer-generated list of random numbers, into one of the three groups: patients in group A received fluconazole mucoadhesive film 20 mg (sustained release) that was to be applied at bedtime and film 10 mg (intermediate release) to be applied during the day after lunch.

**Results:**

There was a significant decrease in oral discomfort, pain and clinical improvement in group A compared to group B (Fluconazole oral tablets 100 mg/day) (P = 0.005) and group C (Fluconazole Mouth rinse) (P = 0.002). The patients who received the mucoadhesive patches had a more tolerable safety profile as expected compared to the other groups.

**Conclusion:**

The bioadhesive films of fluconazole were used in HIV positive patients with oral candidiasis to overcome the problems of high dose requirement of the drug and reduce associated adverse reactions in an already immunocompromised patients and improve the quality of life.

## Plain Language Summary

*Treating Oral Fungal infections in the mouth using Mucobioadhesive patch that contains a strong antifungal infection preventing complications and also improving patients symptoms and quality of life*

Fungal infections in the mouth can be disabling to HIV positive patients as it presents with severe burning sensation and intolerance to spicy food. As a part of treating this fungal infection named oral candidiasis an antifungal medication is usually presribed, either for topical application or for oral intake. If any systemic medication is given, it causes other side effects in an already immunocompromised person, hence, a new formulation is used called Mucobioadhesive patch that contains a strong antifungal action preventing complications and also improving patients symptoms and quality of life.

## Introduction

Oropharyngeal candidiasis remains the most common opportunistic fungal infection among patients with human immunodeficiency virus (HIV) and is often the initial manifestation of HIV infection.^1^ Approximately 80% to 90% of HIV-infected patients develop oropharyngeal candidiasis at some time during the progression of their disease.^2^ Genotyping of candida strains obtained from HIV-infected patients with either oropharyngeal or esophageal candidiasis indicate a genotype distribution frequency like that seen in non-HIV-infected patients, suggesting that HIV-associated mucosal candidiasis is not caused by a unique or particularly virulent strain of Candida, but likely results from defects in host defense mechanisms.^3^

Fluconazole is a triazole antifungal drug that inhibits cytochrome P450-dependent enzymes, resulting in impaired ergosterol synthesis in fungal cell membranes.^4^ Fluconazole is well absorbed after oral doses and is less likely to cause symptomatic relapses compared with topical antifungals^5^ however, systemic fluconazole is associated with adverse effects, such as hepatotoxicity, toxic epidermal necrolysis, leucopenia, etc^6^ Being a CYP3A4 and 2C9 inhibitor, fluconazole is also associated with a number of drug interactions. Considering that fluconazole is the preferred drug in the treatment of oropharyngeal candidiasis^7^ but it is associated with potentially serious adverse effects and drug interactions, especially in patients with HIV,^8^ although there are fewer interactions compared to other systemic antifungals, it is necessary to develop a new fluconazole drug delivery system for topical therapy of oropharyngeal candiasis in HIV patients. Topical therapy is also relevant because pathogenic yeasts in oral candidiasis are usually detected in the superficial layers of the oral mucosa^9^

Within the oral mucosal cavity, delivery of drugs can be achieved by buccal delivery, which is drug administration through mucosal membranes lining the buccal cavity. One of the major limitations associated with the buccal route of drug administration is the lack of retention of the dosage form at the absorption site.^10^ Sholapurkar et al (2009)^11^ evaluated a fluconazole mouth rinse formulation, comparing it with clotrimazole mouth paint; although effective, the drawback of the mouth paint and mouth rinse is the short retention time of the drug. Consequently, bioadhesive polymers have been extensively employed in buccal drug delivery systems in the form of adhesive patches, adhesive films, adhesive tablets, and buccal gels.^12^ Buccal films are highly flexible and ensure more accurate dosing of the drug compared to gels and ointments. Moreover, buccal films are suitable for protecting wound surfaces, thus reducing pain and increasing the treatment effectiveness.^13^ This study aimed to develop a mucoadhesive formulation of fluconazole as oral patches and determine its efficacy and safety in comparison with fluconazole tablet and oral suspension for oral candidiasis in patients with HIV infection. If it was found to be effective, the mucoadhesive formulation would ensure the continued presence of the drug in the oral cavity for the required duration, obviating the potential systemic adverse effects and the chances of drug-drug interactions.

## Material and Methods

### Preparation of Fluconazole Mucoadhesive Films

The fluconazole mucoadhesive films were prepared at the Department of Pharmaceutics, of our constituent institute. The solvent casting technique was used.^14^ For the 20 mg films, weighed quantity of film forming polymers, 2% hydroxypropyl methylcellulose (HPMC; sustained release grade), 2% ethyl cellulose, and 0.5% carbopol were gradually added to the required amount of methanol-water mixture with constant stirring. The choice of the polymers is based on based on the ideal release kinetics and enhanced bio adhesiveness. The required quantity of fluconazole was dissolved in minimum volume of solvent mixture and added to the prepared polymer solution. Propylene glycol was used as the plasticiser. The medicated gel was left overnight at room temperature to ensure clear, bubble-free gel. The gel was cast into a glass Petri dish and allowed to dry at room temperature for the first 2 h and in a vacuum oven at 45 °C for next 4 h, until a flexible film was formed. The dried film was cut into 11 mm diameter circles containing 20 mg of fluconazole, packed in an aluminium foil, and stored in glass containers at room temperature. For the 10 mg films, 5% HPMC (5LV grade) and 1% carbopol were used. The dried film was cut into circles of 9 mm diameter containing 10 mg fluconazole. The fluconazole mucoadhesive films were evaluated for swelling index, film thickness, content uniformity of film, surface pH, percentage moisture absorption, percentage moisture loss, tensile strength of film and backing membrane, folding endurance, *ex vivo* bioadhesive strength, *in vitro* residence time, *in vivo* residence time of placebo buccal patch, validation of impermeability of backing membrane, and *in vitro* release study.

### Study Design and Patients

A randomized, single-blind, active control, clinical trial was conducted among patients with oropharyngeal candidiasis visiting the Department of General Medicine of our Teaching hospitals and antiretroviral treatment centre who were invited to participate in the study. The study protocol was approved by the Institutional Ethics Committee of our institute. Written informed consent was obtained from all study participants. The study was conducted in accordance with the World Medical Association Declaration of Helsinki and Indian Council of Medical Research National Ethical Guidelines for Biomedical and Health Research Involving Human Participants.

The inclusion criteria were as follows: patients of either gender; aged ≥18 years; HIV positive with oral/oropharyngeal candidiasis; CD4 count > 200 cells/µL; not on treatment with any topical or systemic antifungal drugs for more than two consecutive days in the past 2 weeks prior to screening; negative urine pregnancy test in the case of women of childbearing potential; able to understand written and/or verbal instructions to comply with all study requirements; and willing to participate and give written informed consent. Exclusion criteria were patients taking barbiturates or oral anticoagulants; those with known hypersensitivity to the azole group of antifungals; history of alcoholism, drug abuse, psychiatric disorder, or any other problem that can invalidate informed consent; symptoms of oesophageal or systemic candidiasis; severe systemic illness with a life expectancy less than 4 weeks; known liver impairment; women with potential to have children who are unwilling or unable to use an acceptable method of birth control to avoid pregnancy during the study period.

The patients were randomised, using a computer-generated list of random numbers, into one of the three groups: patients in group A received fluconazole mucoadhesive film 20 mg (sustained release) that was to be applied at bedtime and film 10 mg (intermediate release) to be applied during the day after lunch. The patients were instructed to brush their teeth and place the film on the vestibule, between the cheeks and the gingiva, in the upper canine region, applying slight pressure for 60 s. The application of the medicated film was demonstrated to the patients during the first visit. Patients in group B received fluconazole 100 mg tablets, to be taken orally once a day after breakfast. Those in group C received fluconazole mouth rinse 2 mg /mL and were instructed to swish 7.5 mL of suspension and swallow, three times a day after meals, and not eat or drink anything for 2 h after rinsing. The duration of treatment was 14 days for all the groups. The patients were instructed to come for the next visit according to the schedule and report to the physician anytime in case of an adverse event.

### Study Procedures

The study consisted of three visits: Visit 1 (Day 1), clinical examination, swab for mycology culture, dispensing of medication; Visit 2 (Day 7), assessment of clinical improvement and adverse effects, if any; Visit 3 (Day 14), clinical examination and swab for assessment of mycological cure.

Clinical- The clinical symptoms with which the patients presented were graded into mild, moderate and severe and scored as 1, 2 and 3 respectively. The clinical signs with which the patients presented were assessed by the Physicians’ assessment scale and the severity was graded as mild, moderate and severe and scored as 1, 2 and 3 respectively.^15^ For the mycological assessment, in symptomatic patients, oral swabs were obtained by firmly swabbing the lesion site with sterile cotton swabs, and in asymptomatic patients, the dorsum of tongue and buccal mucosa was swabbed; the swabs were immediately dipped in 1 mL of saline.

Laboratory- The samples were immediately inoculated on Sabourauds’ dextrose agar with chloramphenicol (0.05 g/L) and incubated at 37 °C for 2 days and for additional 7 days at 30 °C before being considered as negative. Fungal colony counts and species identification were performed using chrome agar.

### Statistical Analysis

The data was entered into a Microsoft Excel file and data analysis was performed using the Statistical Package for Social Sciences version 11.5 (Chicago, IL, USA). A one-way analysis of variance was used to compare age and CD4 count between groups. Fisher's exact test was used for categorical data. The distribution of data was assessed using Shapiro-Wilk test. Since the data were not normally distributed (p < 0.01, except age, p = 0.570), Mann-Whitney U test was used for multiple comparisons of clinical cure, visual analog scale score, and mycological cure. Kruskal Wallis test was used for between-group comparisons of the clinical cure, visual analog scale score, mycological cure, and adverse effects. Wilcoxon's signed rank sum test was used for within group comparisons of the clinical cure, visual analog scale score, and mycological cure. A p-value <0.05 was considered statistically significant.

With 95% confidence level, 80% power, and effect size of 0.5, the sample size required per group is about 15; considering 30% dropouts, a sample size of 20 patients per group was considered.

## Results

Fifty-five patients with HIV-related oral candidiasis participated in the study; 18 patients in group A (fluconazole mucoadhesive film), 18 in group B (fluconazole tablet), and 19 in group C (fluconazole mouth rinse). One patient did not return for follow-up in group A; one from group B was withdrawn from the study due to severe illness; all patients in group C completed the study. Of the 55 patients, 30 (54.5%) were males. The baseline characteristics of the study participants are shown in Table 1.

**Table 1.** Baseline characteristics of study participants with HIV-related oral candidiasis.

Parameter	Group A (Fluconazole mucoadhesive film) N = 18	Group B (Fluconazole tablet) N = 18	Group C (Fluconazole mouth rinse) N = 19	P-value
Age	40 (35-48)	45 (38-52)	45 (38-48)	0.566
Male	10 (55.6%)	11 (61.1%)	9 (47.4%)	0.699
CD4	300 (250-345)	311 (236-355)	245 (212-370)	0.590

The comparison of the clinical symptoms severity scores, before and after treatment, is shown in Table 2. A significant decrease in the severity of clinical symptoms was observed after treatment in all groups. Out of 18 patients in Group A 70.58% of the patients had clinical resolution and 72% of the patients had mycological eradication. Out of 18 patients in Group B 47% of the patients had clinical resolution and 52% of the patients had mycological eradication. Out of 19 patients in Group C 27% of the patients has clinical resolution and 47.36% of the patients had mycological eradication.

**Table 2.** Within group comparison of the clinical signs and symptoms of oral candidiasis before and after treatment with different formulations of fluconazole.

Clinical Symptoms^a^	Before treatment	After treatment	P-value*
Group A (Fluconazole mucoadhesive film) N = 18	2.0 (2-3)	0.0 (0-0)	<0.001
Group B (Fluconazole tablet) N = 18	2.5 (2-3)	0.0 (0-1)	<0.001
Group C (Fluconazole mouth rinse) N = 19	2.0 (2-2)	1.0 (1-1)	<0.001
Visual analogue scale score for oral discomfort ^b^	Before treatment	After treatment	P-value*
Group A (Fluconazole mucoadhesive film) N = 18	7 (5.75-8)	0 (0-1)	<0.0001
Group B (Fluconazole tablet) N = 18	7 (5.75-8)	1 (0-3)	<0.0001
Group C (Fluconazole mouth rinse) N = 19	7 (6-8)	2 (0-3)	<0.0001
Clinical signs^c^	Before treatment	After treatment	P-value*
Group A (Fluconazole mucoadhesive film) N = 18	2.0 (2-3)	0.0 (0-1)	<0.001
Group B (Fluconazole tablet) N = 18	3.0 (2-3)	1.0 (0-1)	<0.001
Group C (Fluconazole mouth rinse) N = 19	2.0 (2-3)	1.0 (1-1)	<0.001

a. The clinical symptoms with which the patients presented were graded into mild, moderate and severe and scored as 1, 2 and 3 respectively

b. VISUAL ANALOG SCALE: Assessment of oral discomfort (burning sensation, pain) by the patient ranging from 0 to 10, 0 being the lowest and 10 the maximum.

c. The evaluation of oral candidiasis was performed subjectively, by the clinician.

Mild: Affects a single area of the oral cavity

Moderate: Affects multiple areas, such as the tongue and buccal mucosa

Severe: Affects numerous areas, including the tongue, buccal mucosa, palate, and floor of the mouth

*Wilcoxon signed rank test

There was a significant difference in the magnitude of improvement in symptoms between the groups (KW = 18.90; P < 0.001); patients in group A and group B had a greater improvement in symptoms compared to group C (P < 0.001 and P = 0.002, respectively). There were no significant differences in the improvement of symptoms between groups A and B (P = 0.247).

Table 2. Within group comparison of the clinical signs and symptoms of oral candidiasis before and after treatment with different formulations of fluconazole There was a significant decrease in oral discomfort, as determined using VAS, after treatment in all groups.^2^ A significant difference in VAS scores was observed between the groups (KW = 11.585; P = 0.003); patients in group A experienced a greater improvement in oral discomfort compared to group B (P = 0.005) and group C (P = 0.002).

There was a significant decrease in the clinical signs, as determined using the Physicians Assessment Scale, following treatment in all the groups (Table 2 A significant difference was observed in the improvement of clinical signs between the groups (KW = 14.203; P < 0.001); groups A and B had a greater improvement compared to group C (P = 0.001 and P = 0.005, respectively); there were no significant differences between groups A and B (P = 0.754).

There was a significant decrease in colony count after treatment in all groups (Table 3). There was no overall difference in the magnitude of improvement of colony count between the groups (KW = 3.804; P = 0.149). On comparison of the candida species after treatment in the three groups, the percentage of resistant species, like *C. glabrata* and *C. krusei*, was more in groups B and Group C compared with group A. Mycological cure was observed in 72%, 52.94%, and 47.36% patients in groups A, B and C, respectively.

**Table 3.** Within group comparison of the colony count before and after treatment of oral candidiasis with different formulations of fluconazole.

	Before treatment (Mean)	After treatment (Mean)	P-value
Group A (Fluconazole mucoadhesive film) N = 18	1825	23.44	<0.001
Group B (Fluconazole tablet) N = 18	1727.78	133.82	<0.001
Group C (Fluconazole mouth rinse) N = 19	1896.84	226.28	<0.001

Mycological cure defined as a nil colony count after treatment, was observed in 72%, 52.94%, and 47.36% patients in groups A, B and C, respectively.

Table 3. Within group comparison of the colony count before and after treatment of oral candidiasis with different formulations of fluconazole.

The three treatment regimens were well tolerated. There were no adverse effects seen in Group A. In Group B there were 2 cases reported with elevated liver enzymes, 6 patients with GI discomfort and one patient died due to encephalitis. In Group C there were 2 cases of elevated liver enzymes and 7 cases of GI discomfort reported Table 4.

**Table 4.** Candida species isolated from study participants before and after treatment.

	A		B		C		Total	
	Before	Afterward	Before	Afterward	Before	Afterward	Before	Afterward
No organism	0	1	0	7	0	6	0	14
C.albicans	11	1	8	1	5	2	24	4
C.krusei	1	1	0	1	0	5	1	7
C.glabrata	0	1	0	7	0	3	0	0
C.tropicalis	1	1	0	1	0	0	1	2
C.albicans + C.glabrata	2	0	3	0	7	0	12	0
C.albican + C.krusei	1	0	1	1	3	0	5	1
C.krusei + C.glabrata	0	0	1	0	0	2	1	2
C.albicans + C.tropicalis	1	0	1	0	0	0	2	0
C.albicans + C.krusei+ C.glabrata	1	0	4	0	4	0	9	0
Total	18	5	18	18	19	18	55	41

## Discussion

We compared the newly developed fluconazole mucoadhesive patches to fluconazole oral rinse and pills as usual treatments. The corresponding clinical resolution rates were 70.58%, 47% and 27% with Group A, B and C respectively. Antifungal gels or suspensions can also be used to treat oral candidiasis; however, the release of medication from these formulations is characterized by an early burst of action, which is quickly followed by a rapid drop of the local drug concentration to subtherapeutic levels.^16^ Thus, for the treatment of oral candidiasis, systemic antifungals like fluconazole are typically recommended.^17,18^ Using 100 mg fluconazole pills as treatment, Koletar et al found a 100% clinical remission rate in their preliminary study of 39 patients with oral candidiasis and HIV infection.^19^

In 69 individuals with oral candidiasis, a study by Sholapurkar et al^11^ using an oral rinse containing 30 mg fluconazole demonstrated a 96.29% clinical remission rate. The physicians’ assessment scale used in our study had a strict criterion for evaluating resolution; if even mild erythema was present, the score was 1, not indicating complete resolution of the disease. This could be the reason for the variation in the clinical resolution rate that we observed. The fact that the individuals in our study have impaired immune systems may also be a contributing factor.

In contrast to our study, which found 52.94% mycological eradication, the Koletar et al^19^ study claimed 75% mycological eradication in the group that took 100 mg of fluconazole tablets. The significant variation in mycological cure rates may result from the use of distinct sample and cultured techniques. In our investigation, the swab was directly inoculated on Sabouraud Dextrose Agar, following a typical protocol for candida culture, rather than being dipped in 1% saline. To buy some time before the inoculation, dipping the swab in saline could result in a falsely low candida colony count. Furthermore, the disparity in cure rates could have been influenced by the growing resistance to fluconazole. In the oral rinse group, the mycological eradication was 47.36%, whereas in the Sholapurkar research^11^ it was 88.88%.

The patients who received the mucoadhesive patches had a tolerable safety profile compared to the systemic administrations. Two occurrences of increased liver enzymes in patients receiving tablets and mouth rinse were documented. Six occurrences of gastrointestinal distress were recorded in the group that took fluconazole tablets, and seven cases in the group that used oral rinses.

Compared to traditional formulations, the fluconazole mucoadhesive patch offers a number of advantages. First, oral candidiasis associated with HIV frequently causes dry mouth. When patients take fluconazole pills for therapy, it's possible that the salivary concentration of the drug won't rise above the minimum inhibitory concentration (MIC), which could result in treatment failure or recurrent episodes brought on by the resistant species. This disadvantage is addressed by fluconazole mucoadhesive patches, which operate locally at the targeted site of action.^20^ Regarding the time course of effect, the second benefit is that fluconazole is detectable in saliva two hours following systemic dose is administered making it locally available at the site of action.^5,21,22^ Fluconazole mouthwash also improves the drug's immediate and four-hour exposure to the oral mucosa as shown in other studies^11^

Our trial was not double-blinded and hence a possibility of bias may be there and follow up visits were not well standardised can be a potential demerit to this study.

## Conclusions

Oral candidiasis known for its opportunistic role in immunocompromised patients is usually difficult to treat until the treatment is tailor made for local action. Topical treatments with fluconazole show a similar efficacy as systemic effect when delivered at the site. This mode of administration also reduces drug interactions and adverse events and is well tolerated. The results of this study can be confirmed by a larger study to establish the efficacy and safety of fluconazole mucoadhesive patches compared to other standard treatments.
